# Supplementary figures and images for: Chromosome-Level Genome Assembly and Population Genomic Analyses Reveal Geographic Variation and Population Genetic Structure of Prunus tenella
Source: Int J Mol Sci. 2023 Jul 21;24(14):11735. doi: 10.3390/ijms241411735 (PMC10380494; doi:10.3390/ijms241411735)

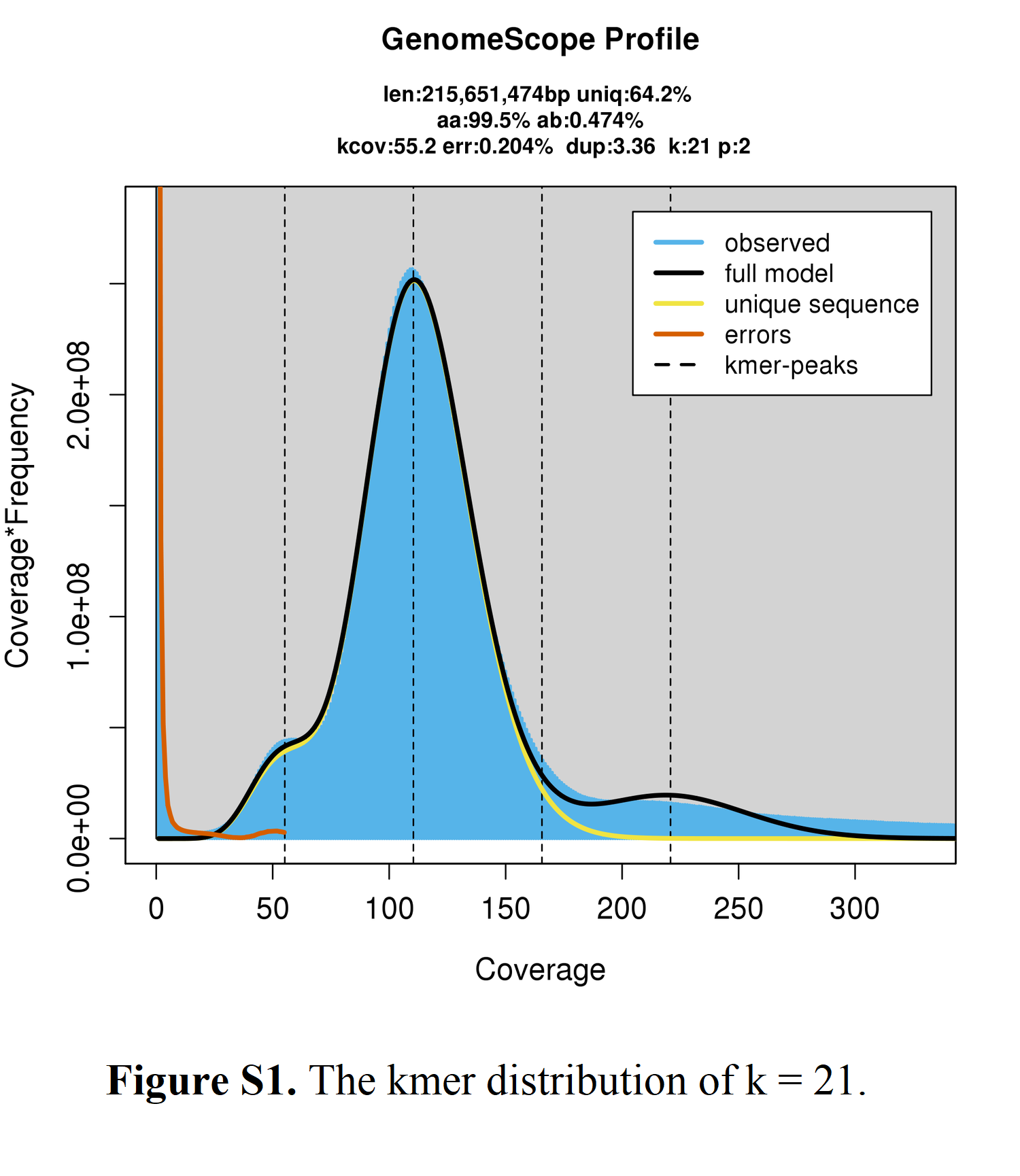

Supplement: Supplementary file 1 [file ijms-24-11735-s001.zip › Supplementary Figure S1.png]
